# Forbidden versus permitted interactions: Disentangling processes from patterns in ecological network analysis

**DOI:** 10.1002/ece3.3102

**Published:** 2017-06-13

**Authors:** Giovanni Strona, Joseph A. Veech

**Affiliations:** ^1^ European Commission Joint Research Centre Directorate D ‐ Sustainable Resources – Bio‐Economy Unit Ispra Italy; ^2^ Department of Biology Texas State University San Marcos TX USA

**Keywords:** artificial life, co‐occurrence, ecological networks, food web, functional traits, mutualism, nestedness, pollinators

## Abstract

Several studies have identified the tendency for species to share interacting partners as a key property to the functioning and stability of ecological networks. However, assessing this pattern has proved challenging in several regards, such as finding proper metrics to assess node overlap (sharing), and using robust null modeling to disentangle significance from randomness. Here, we bring attention to an additional, largely neglected challenge in assessing species’ tendency to share interacting partners. In particular, we discuss and illustrate with two different case studies how identifying the set of “permitted” interactions for a given species (i.e. interactions that are not impeded, e.g. by lack of functional trait compatibility) is paramount to understand the ecological and co‐evolutionary processes at the basis of node overlap and segregation patterns.

## 1 INTRODUCTION

Mapping the links connecting resources to consumers in complex networks of species interactions has become a fundamental approach in community ecology. Several studies have suggested that the structure of such networks provides a key to improve our understanding of how complexity emerges and is maintained in natural systems. Among the different network properties, much interest has been dedicated to the tendency of species toward sharing interacting partners either more or less than expected at random. In particular, the tendency for sharing more partners than expected, a pattern known as “nestedness” (Ulrich, Almeida‐Neto, & Gotelli, 2009), has been suggested as ubiquitous in ecological mutualistic networks (Bascompte, Jordano, Melián, & Olesen, 2003), and possibly beneficial to their persistence (Bastolla et al., 2009; Rohr, Saavedra, & Bascompte, 2014; but see also James, Pitchford, & Plank, 2012; Allesina & Tang, 2012).

However, assessing how much the tendency to share partners departs from randomness has proved challenging, both for the identification of a proper metric, and of a robust null model capable of disentangling significance from randomness. In an attempt to contribute to the discussion, Strona and Veech (2015) have developed a new measure $\overset{¯}{N}$capable of identifying patterns varying from node overlap to the opposite tendency (i.e. node segregation). Among available metrics, $\overset{¯}{N}$ has some particular characteristics that make it extremely flexible, and capable of being applied to any kind of network –not only bipartite networks such as plant pollinators, but also unimode networks such as food webs, despite recent unfounded criticism stating the contrary (Chen, 2016; Appendix S1).

The procedure by Strona and Veech (2015) consists in deriving by averaging a standardized measure of pairwise node overlap (Ɲ_ij_) obtained, for each pair of nodes (i.e. species) *i* and *j* by comparing the observed number of shared partners, to that expected according to basic probability and combinatorics. The expected number of shared nodes takes into account both the respective numbers of interacting partners for each of the focal nodes, and the total number of partners that the two nodes can possibly (realistically) share. This latter value (corresponding to the parameter *n* in Strona & Veech, 2015, eqn. 2) must be specified within the limits of ecologically plausible interactions.

In absence of any particular assumption about plausible interactions, one may specify the sets of potentially shareable partners for each node (and hence *n* for each pair of nodes), based solely on the number of available species in the network. In the bipartite case, for example, a plant‐pollinator network, a naïve specification of *n* is fairly straightforward. Thus, when computing the pairwise node overlap (Ɲ_ij_) for two pollinator species, *n* could be set equal to the total number of plant species in the network. Indeed, this would be the correct approach if the researcher had no a priori information about the functional and structural compatibility of each pollinator species with each plant species. However, sometimes relevant and useful ecological information is available. The Ɲ_ij_ measure of Strona and Veech (2015) allows for the application of explicit rules aimed at identifying specific sets of shareable nodes by including or excluding potential interacting partners on the basis of general or specific criteria. Further, applying explicit rules for identifying potential interacting partners may often be a critical element in testing specific hypotheses.

For instance, when computing $\overset{¯}{N}$ in a food web, one can either or not account for cannibalistic behaviors, which correspond to loops in a network (i.e. nodes linked to themselves). In the latter case, in each pair comparison, one should exclude the two focal nodes in the computation of Ɲ_ij_, hence assuming that a resource will never consume itself, by replacing the parameter *n* with (*n*−2). In addition, or in alternative, one may identify smaller sets of partners possibly shared by the two focal nodes, and hence smaller values of *n* on the basis of particular ecological criteria. For example, using functional traits, one may limit the set of possible partners for two pollinator species to the plant species whose pollen is physically (i.e. mechanically) accessible to both pollinators. We note that respecifying *n* (from all nodes in the network to a smaller restricted set) does not alter the basic assumption that each node in the specified set of potential partners has an equal probability of being linked to the focal pair. The equal probability assumption is a feature of the hypergeometric sampling distribution, which is the foundation of our approach (Strona & Veech, 2015).

This particular feature makes $\overset{¯}{N}$ a very flexible tool to test a broad range of hypotheses. Furthermore, it poses a fundamental question about our current perception of node overlap (species sharing) in ecological networks, and the way ecologists have evaluated it to date. Commonly used nestedness metrics, such as the very popular NODF (Almeida‐Neto, Guimaraes, Guimarães, Loyola, & Ulrich, 2008), have been conceived for bipartite networks, that is, networks where nodes belong to two distinct categories, and where interactions do not occur between nodes belonging to the same category. Such metrics are based on the underlying assumption that a node (e.g. a species) can potentially interact with all the members of the other category (i.e. the assumption that a flowering plant species can be pollinated by all pollinating species in the network, or that a parasite can infect all available hosts).

**Figure 1:**
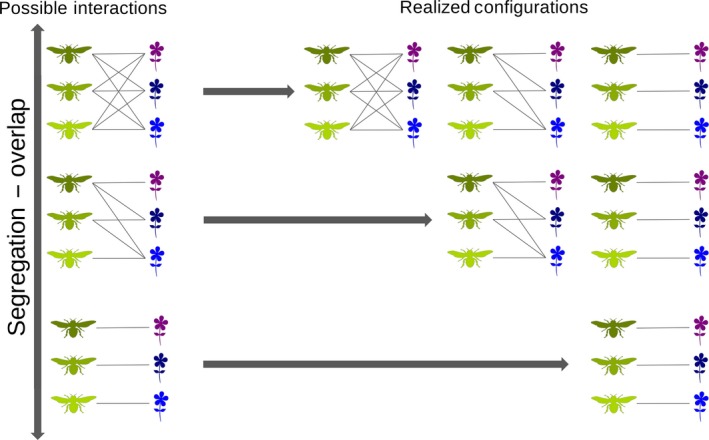
The set of possible pairwise interactions constrains the range of realized network configurations

Here, we demonstrate that this assumption could complicate the interpretation of observed patterns, because the range of possible interactions could be subject to various constraints due, for example, to co‐evolutionary processes. For instance, the co‐adaptation between proboscis length of moths and nectar position in the flowers of different orchid species (Micheneau, Johnson, & Fay, 2009) clearly limits the number of shared pollinators for each pair of orchid species and vice versa. In such a scenario, a situation of high node segregation could emerge either because species do not have the potential to interact with common partners, or due to ecological processes (such as competition) forcing them to use different resources (Figure 1). Assume that in a large network two different pollinators are observed visiting only one (the same) plant species. It would be clearly important to understand if the target plant is actually the only species accessible to the two pollinators, or if the overlap in resource use should be attributed to other reasons potentially of greater ecological interest (possibly, some beneficial interaction between the two pollinators).

Due to the wide range of possible factors limiting/controlling the accessibility to a resource (e.g. synchronicity between plant flowering period and pollinator life cycle, visual and chemical communication), having enough information to identify the true set of possible interacting partners could be extremely challenging, and further complicated by the blurred boundaries between co‐evolutionary constraints and ecological factors. On the one hand, taking into account only a few constraining factors would likely lead to overestimating the “true” number of potential partners. For example, we may end up considering as possible an interaction between a pollinator and a plant with morphological compatibility, but asynchronous life cycles. On the other hand, however, this would still provide a more realistic assessment than just assuming all interactions as possible. Furthermore, performing different analyzes by keeping fixed one factor at a time could be an effective way to identify their relative effects on network structure and hence test various hypotheses.

To demonstrate how using a priori specified subsets of potentially shared partners can deeply affect the results and interpretation of an analysis of network structure, we performed two independent analyzes on both real and simulated networks, showing how the application of different criteria may yield substantially different outcomes. Our results suggest that a more conscious attempt at taking into account permitted vs. forbidden interactions could be the key to a more complete understanding of patterns and processes in ecological networks.

## 2 REAL NETWORKS

We examined the structure of 181 food webs compiled by Cohen (2010). The food webs include several kinds of systems. We classified them into two broad categories, namely 145 persistent and 36 ephemeral food webs. The first included typical food webs, such as large, relatively open systems as lake or forest food webs, while the latter included small systems relying on resources only temporarily available, such as communities living in fallen trees, or in animal corpses. The complete list of food webs and their categorization is provided in Table S1.

**Figure 2:**
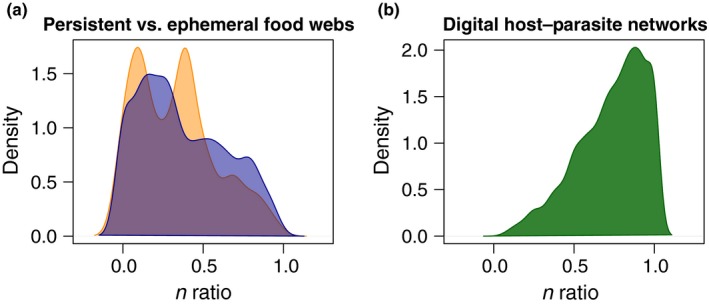
Density plots showing the distribution of the ratios between the size of the set of potential interacting partners for a given node reduced according to specific criteria, and that of the complete set of interacting partners, for all nodes in the respective networks. The sets of potential interacting partners were reduced according to trophic rules in food webs (a; blue = persistent, orange = ephemeral), and according to a task‐matching criterion in digital host parasite networks (b, see Simulated Networks)

Intuitively, one should expect a higher degree of structure in persistent food webs than in ephemeral ones, due to the longer temporal scale, and the likely higher diversity and abundance of ecological structuring processes. We evaluated if this was true, and how much it was affected by considering or not forbidden links, by computing the measure of overlap either assuming that all species can consume other species, or by constraining consumer–resource interactions by adding a rule based on trophic levels. For this, we first identified trophic levels in each network according to Williams and Martinez (2004) as the minimum path distance of target species to basal resource. Then we consider as permitted only links from a resource to a consumer at a higher trophic level. This is still far from being a perfect representation of possible interactions, yet it offers a more realistic picture than considering all links as permitted. Taking into account trophic levels led to a substantial reduction in the set of potential interacting partners (*n*) for each node. On average, the ratios between the size of the complete set of a node's partners (i.e. all nodes in the network) and the corresponding set reduced according to the selected trophic rules were 0.37 and 0.33, respectively, for persistent and ephemeral food webs (Figure 2a).

**Figure 3:**
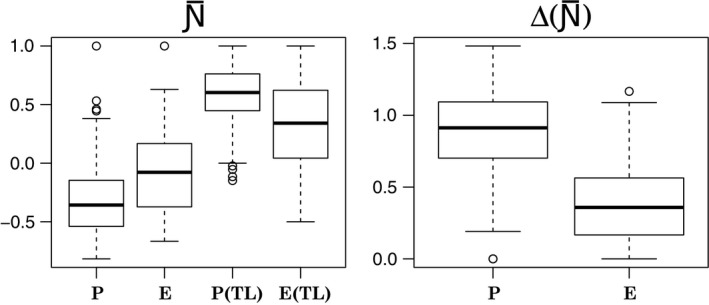
Comparison between node overlap in persistent (P) vs. ephemeral (E) food webs computed either taking into account or not trophic levels (TL). Boxplots in the left panel summarize the $\overset{¯}{N}$ values in the two categories of food webs and in the two different experiments (i.e. with/without trophic constraints). Those in the right panel summarize the difference between the $\overset{¯}{N}$ values computed taking into account trophic levels, and those computed considering all interactions as possible in the two sets of food webs. Boxes indicate first and third quartiles, whiskers indicate range values, horizontal lines indicate median values, and dots indicate outliers

For both persistent and ephemeral food webs, taking into account trophic levels revealed different patterns from those observed when all interspecific interactions were assumed as possible (Figure 3). In the first case, persistent networks showed a moderate tendency for segregation (i.e. less overlap than expected in shared partners), with mean $\overset{¯}{N}$ = −0.31 ± 0.05 (95% *CI*), while ephemeral networks resulted mostly random mean $\overset{¯}{N}$ = −0.03 ± 0.14. But, when trophic levels were taken into account, both sets of networks exhibited a stronger pattern of node overlap that, as expected, were much stronger in persistent networks than in ephemeral ones (with mean $\overset{¯}{N}$ = 0.34 ± 0.13 for ephemeral and $\overset{¯}{N}$ = 0.58 ± 0.04 for persistent webs).

## 3 SIMULATED NETWORKS

We applied our metric ($\overset{¯}{N}$) to digital ecological networks evolved in the artificial life simulation platform Avida (http://avida.devosoft.org/). In Avida, “organisms” compete and replicate themselves in a virtual environment over time (Ofria & Wilke, 2004). The digital organisms interact with the environment by performing different logical operations (referred to as “tasks” in Avida) involving the manipulation of binary strings. The tasks differ in their degree of complexity and, depending on the experimental setting, may provide different benefits to the digital organisms and/or affect their vulnerability toward “parasites.” Parasites are other digital organisms capable of targeting “free‐living” organisms (i.e. hosts) and stealing part or all of their resources (Zaman et al., 2014). Avida simulates competition and, in the long run, evolutionary dynamics very consistent with those observed in natural systems (Fortuna, Zaman, Wagner, & Ofria, 2013; Lenski, Ofria, Pennock, & Adami, 2003).

Here, we used the 100 host/parasite networks generated in Avida by Strona and Lafferty (2016). Those networks, that included from 1 to 65 hosts (mean 19.6 ± 11.8 *SD*), from 2 to 78 parasites (mean 20.1 ± 18.4), and from 3 to 479 interactions (mean 88.8 ± 93.5), have evolved in a broad range of environmental conditions, constitute a perfect benchmark for our test for several reasons. First, different from natural ecological networks, they are fully resolved (i.e. we have complete information on existing hosts, parasites, and their interactions). Second, they include all (and only) the species that have participated in the co‐evolutionary phase. Third, they are built according to a specific, objective rule permitting parasites to infect a host: namely, a parasite can infect a host only if it can perform at least one of the logical operations performed by the host (see Zaman et al., 2014). For example, one of the simplest logical operations performed by an Avidian digital organism is the “NOT” task, where an organism manipulates a 32 bit binary string replacing each 0 with a 1 and *vice versa*. As the complexity of this task is low, it will be easily evolved by parasites, creating a strong selection against hosts performing “NOT,” hence promoting the diffusion of those hosts capable of performing more complex tasks, and giving rise to co‐evolutionary dynamics.

The execution of tasks in Avida needs specific resources (i.e. performing the “NOT” task may require, depending on the setting, different resources than those needed to perform another task). As resources are available in limited quantities, species compete for them. Furthermore, performing a logical operation can lead to the production of other resources. Thus, a host capable of performing a certain task requiring a specific resource, and another task requiring another resource, could be forced by stronger competitors using one of the two resources to not perform one of the two tasks. In turn, this would expose the host to only some of all the parasites that could potentially use it, while protecting it from parasites targeting the unperformed task.

The task‐matching rule makes it possible to identify exactly the set of hosts that could potentially be infected by a given parasite, and the set of parasites that could potentially infect a given host. In turn, this allows us to discriminate precisely between the overlap/segregation due to co‐evolutionary constrains (i.e. the set of abilities to perform logical operations acquired by organisms during the co‐evolutionary/arms race phase), and that due to ecological processes such as the resource competition described above.

We examined node overlap (species sharing) in all of the 100 networks from Strona and Lafferty (2016) first by assuming all species as possible partners of each other, and then by limiting the set of interacting partners only to species having matching tasks. The ratio between the set of potential interacting partners obtained by taking into account task matching and the complete one (i.e. all hosts for a given parasite, and all parasites for a given host) was 0.73 averaged over all host and parasites (Figure 2b). Thus, *n* was not reduced as much for these simulated host–parasite networks as it was for the real food web networks.

**Figure 4:**
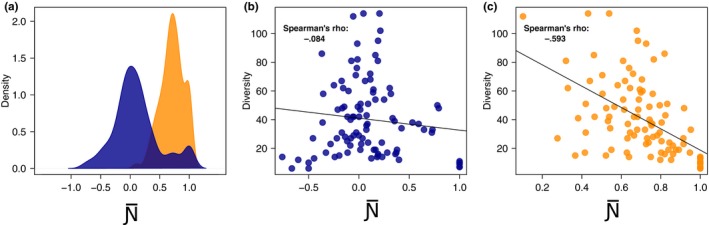
Results of the experiments on digital host–parasite networks. (a) Node overlap computed using all species as potential interactors (blue) and overlap computed when interactions are constrained to a subset of interactors that have a common trait or task (orange). (b, c) the relationship between overlap/segregation and species diversity in the 100 host/parasite digital networks, with overlap measured either assuming (b) all interactions, or (c) the limited set

We found that many networks appear random or even segregated when the overlap is evaluated among all partners (min $\overset{¯}{N}$ = −0.76; max $\overset{¯}{N}$ = 1; mean $\overset{¯}{N}$ = 0.11; CI 95% = ±0.08). However, when the overlap is evaluated under the condition of a constrained (and more realistic) set of potential partners, then the networks show a strong tendency toward overlap/nestedness (min $\overset{¯}{N}$ = 0.10; max $\overset{¯}{N}$ = 1; mean $\overset{¯}{N}$ = 0.71; CI 95% = ±0.04) (Figure 4a).

Without taking into account prior information (such as the species actually capable of being shared by two focal species), we found no relationship between network structure and species diversity (Spearman's rho = −0.08, *p*‐value = .4088 when a constrained set of species is not used). In this scenario, most networks (from species poor to species rich networks) showed randomness in their structure, while only networks with low species diversity showed either overlap or segregation (Figure 4b). When taking into account species actually capable of sharing partners, however, the networks showed more structure, and a clearer, negative relationship between node overlap and species diversity (Spearman's rho = −0.59, *p*–value < .0001) emerged (Figure 4c). This indicates that when diversity is low, parasites tend to use all the hosts that they can access, while in cases of high diversity they tend to differentiate (become selective in) their host usage. This pattern was only revealed by basing the analysis on a constrained set of potential partner species, that is, using a realistic value of *n* in the calculation of $\overset{¯}{N}$.

## 4 DISCUSSION

Ecological networks include a very broad range of different scenarios. The formal distinction made by dividing them into bipartite or unimode, or directed or undirected is a good starting point to discriminate, but it is also obvious that a more fine grained distinction can be made but only on a per case basis. In this study, we showed how our understanding of network structuring processes can be enhanced by taking into account an important, yet largely neglected aspect (at least in the field of network pattern analysis), namely the distinction between forbidden and permitted interactions between network members. The two case studies we presented demonstrate how this adjustment could be fundamental to a proper identification of structural patterns that run the risk of going unnoticed in the dense network of all possible interactions, and to disentangle the effects of co‐evolution from ecological processes in network structural patterns. Those, however, are just a couple of examples of all the possible benefits deriving from making explicit the sets of potential partners two nodes can share.

The choice of two a priori, intuitive starting hypotheses (i.e. the higher structure of persistent systems than ephemeral ones, and the positive relationship between species overlap and community richness) were mostly dictated by our goal of showing the importance of constraining the set of interacting partner species. However, we see a common application of the same approach as particularly suitable to exploratory analyzes aimed at identifying the major determinants of network structure. In particular, one may identify different levels of structure nested within different degrees of constraint. For example, this may lead to the discovery that species tend toward overlap in their use of interaction partners when we impose some ecological constraints, but not when we imposed others. This may reveal, for example, that species appearing not to compete for resources when they are considered as capable of using all resources in an ecosystem are indeed in strong competition when there are environmental constraints on resource use and interactions.

Taking into account ecologically realistic interactions could be helpful for another important issue in the analysis of ecological networks. The common procedure of comparing the observed amount of nestedness with those of a set of null networks created by randomizing the original one according to different rules (Almeida‐Neto et al., 2008; Atmar & Patterson, 1993; Bascompte et al., 2003; Bastolla et al., 2009) is made necessary by the dependence of nestedness measures on network properties. In particular, connectance (i.e. the percentage of realized interactions over the total possible ones) creates an upper boundary for most indices, including the popular NODF (Almeida‐Neto et al., 2008). Null model analysis circumvents this issue and permits identification of nestedness in weakly connected networks because evaluation is not based on absolute NODF values but on their comparison with those computed in randomly rearranged versions of the observed network (having the same connectance).

However, viewing connectance as a network property that should be controlled when measuring nestedness could limit our understanding of the determinants of network structure. In particular, this may lead us to consider as equally structured (in terms of nestedness) two networks having very different connectance, thus ignoring the possibility that the frequency of ecological interactions (i.e. connectance) may reflect the action of ecological processes just as does nestedness. If the fraction of realized interactions in a network is small (low connectance), then perhaps the tendency of nodes to share mutualistic partners (i.e. the mechanism suggested to promote network stability by reducing the overall competitive load, see Bastolla et al., 2009; Jonhson, Domínguez‐García, & Muñoz, 2013) is not so strong and/or widespread across the network.

As an example, one of the largest known pollinator networks consists of more than 15,000 documented interactions between 1,429 animal species visiting flowers of 456 plant species in a small area in southwestern Illinois, USA (Memmott, Craze, Waser, & Price, 2007). This network has a connectance of ~2%. Almost a third of pollinators present in the Illinois network pollinate only one plant species, and 87% of pollinator species pollinate less than 5% of available plant species. Similarly, 86% of plant species in the network can be pollinated by less than 5% of available pollinator species. It is noteworthy that the average number of plant partners shared by two pollinator species is less than one (0.69), making it difficult to infer a widespread tendency for sharing mutualistic partners. Actually, this issue extends to most of the mutualistic networks that have been investigated to date (all available at http://www.web-of-life.es/), and that have a very low connectance (on average <7%), which supports the idea that specialization of interactions (and not nestedness) may promote the co‐existence of multiple species (Pauw, 2013).

Identifying permitted interactions between species, and focusing on those only to assess species’ tendency to share partners could be a promising approach to shed light on this issue. The empty cells in a presence–absence adjacency matrix representing an ecological network contain both ecological and co‐evolutionary information. Both are useful to improve our understanding on the functioning of complex systems, but traditional approaches do not offer straightforward ways to disentangle the first from the latter, leading to a unique–yet hardly interpretable–result. Conversely, the alternative approach we have discussed here analyzes the structure of a network from different angles, providing specific answers to properly defined hypotheses.

We do not question the importance of comparative studies trying to identify general patterns in ecology. However, we also think that the current ecological debate on the mechanisms promoting and regulating the complex structure of ecological network could benefit also from more targeted studies designed on a per case basis. We hope that this paper can be a step in that direction.

## CONFLICT OF INTEREST

None declared.
