# Supplementary material for: Forbidden versus permitted interactions: Disentangling processes from patterns in ecological network analysis
Source: Ecol Evol. 2017 Jun 13;7(14):5476–81. doi: 10.1002/ece3.3102 (PMC5528246; doi:10.1002/ece3.3102)
Supplement: Supplementary file 1 [file ECE3-7-5476-s001.pdf]

Appendix S1. Node overlap and segregation measure ($\bar{N}$) can be applied to any kind of network

In a recent letter, Chen (2016) (hereafter 'Chen') argued that the measure of network structure ($\bar{N}$) introduced by Strona and Veech (2015) (hereafter 'SV') cannot be applied to non-bipartite networks, such as food webs. Most of Chen's criticism revolves around potential issues regarding the parameter *n* in SV equation [2]. This parameter indicates the total number of partners that two nodes can possibly share. In the bipartite case, such as in a plant-pollinator network, a naïve identification of *n* is fairly straightforward. For example, when computing the node overlap (N_{ij}) for two pollinators, *n* could be set equal to the total number of plants in the network. However, one may identify smaller sets of partners possibly shared by the two focal nodes, and hence smaller values of *n* on the basis of different criteria. For example, SV clarify that, when computing $\bar{N}$ in a food web, one can either or not take into account cannibalistic behaviors (Fox 1975), which correspond to loops in a network (i.e. nodes linked to themselves). In the latter case, SV explain that, in each pair comparison, one should exclude the two focal nodes from the computation of N_{ij}, and hence replace the parameter *n* with (*n* - 2). Noteworthy, most of Chen's criticism can be summarized by his opinion that the n-2 adjustment should always be made. However, Chen's equation [6] is simply a particular case of the more general equation [2] given by SV.

Nevertheless, SV make explicit that the case of cannibalistic interaction is just an example, and that users are perfectly free to identify the sets of shareable partners (and thus adjust *n*) according to specific hypotheses: "This derives directly from network structure, however, specific rules can be applied on a per case basis to improve ecological realism of $\bar{N}$, by identifying a particular set of nodes to be used in the computation of each N_{ij} value (adjusting n as the size of that set)" (p. 909 in SV). For example, using functional traits, one may limit the set of possible partners for two pollinators to the plants whose pollen is physically (e.g. mechanically) accessible to both pollinators.

This particular feature of $\bar{N}$, provides ecologists with a very flexible tool to test a broad range of hypotheses. Contrary to this, Chen's criticism arises from the opinion that *n* is or should be a fixed value derivable from network structure and identified, in all of his examples, as the number of species in the network rather than the number of partners that two nodes can share (which is not necessarily the same for any two nodes).

Of course, general criteria to identify the maximum set of shareable partners (and hence *n*) for two nodes

regardless of specific hypotheses need to be provided. Despite Chen's claims, the criteria provided by SV are correct, and the 'errors' pointed out by Chen derive from his misinterpretation of some basic network properties. The two alternate network configurations presented by Chen in his Fig. 1A and Fig. 1B actually belong to two different networks. In fact, the degree of each node in Fig. 1A is equal to 1, while the degree of nodes in Fig. 1B is 2, doubling the overall network degree (loops always have degree equal to 2, Gross & Yellen 2005, pp. 8-9).

Thus, in this specific case, the adjustment originally suggested by SV is necessary, i.e. the two nodes should be excluded from the computation of overlap probability, and *n* should be replaced by *n*-2 (SV, p. 909). Consequently, SV's equation will give the same probabilities given by Chen (despite his incorrect representation of possible configurations for the two-nodes network). In particular, the exclusion of the two nodes from the computation and the adjustment of *n* to *n*-2 will give (according to SV) $p(k|n,d1,d2) = p(k,0,0,0)$, that will be equal to 1 for k=0, and equal to 0 for k=1.

The situation is different for a directed network such as that in Chen's Fig. 2. SV clearly stated that N_{ij} must be evaluated separately for in and out-going links. This means that when considering outgoing links, one needs to account for alternative configurations of the network independently from how these would affect node in-degree. Thus, the probability of node sharing is evaluated focusing just on the in- or on the out-degree of nodes separately, meaning that, when focusing on incoming links, all the configurations keeping the in-degree (but not necessarily the out-degree) should be considered (and vice versa when considering outgoing links).

Chen briefly mentions in his conclusions that this approach could be a simple solution to the fact "the probability of observing k shared neighbors for a pair of species in directed networks with self-loops "may be more complicated because of the involvement of directions. It should be related to indegree and outdegree of nodes". Indeed, the separate computation of $\overline{\mathrm{N}}_{in}$ and $\overline{\mathrm{N}}_{out}$ described in SV is a key aspect of the method, not only from a computational perspective, but also from a conceptual one. It is intuitive that the overlap in plant species used by two pollinator species is fundamentally different from the overlap in pollinators servicing two plant species. But the same is true also for non-bipartite networks, due to the obvious differences in the processes represented by edge direction (e.g., consuming or being consumed in food-webs, buying or selling in trade networks, departing or arriving in transport networks) (Newman & Park 2003).

Separating in- and out-degree, the possible configurations of the directed network in Chen's Fig. 2 are those we show here in Fig. S1, which makes the probability of 0.5 given by SV's formula correct. Completing the computation of $\overline{\mathrm{N}}$ for the directed network of Fig. 2, we will have:

$\omega_{12} = (0 - 0.5)/min(1,1) = -0.5$

Since $d1 + d2 - n = 0$

then:

$\Omega_{12} = (0.5 - 0)/1 = 0.5$

and hence:

$$\mathrm{N}_{out_{1,2}} = -\frac{0.5}{0.5} = -1$$

The same applies to $\mathrm{N}_{in_{1,2}}$. Therefore, we have $\overline{\mathrm{N}}$ = -1, which makes sense, given that the network is completely segregated. Note that $\overline{\mathrm{N}}$ would have been equal to 1 for a perfectly nested network, such as one of the alternative configurations with one node shared, for example {(1,2); (2,2)}.

Interestingly, Chen applies the SV method correctly to bipartite networks of species distributed among sites as in his Fig. 3. For the overlap of sites across species, he obtains a probability value of overlap identical to that resulting from SV's approach. However, for species-site networks, focusing only on species constitutes just half of the computation of network structure. Chen seems to consider the overlap in species per site as irrelevant.

As an example to verify the correctness of his equations, Chen computes the probability of sharing 0 or 1 nodes for two nodes having degree 1 and 2 on a network having 4 nodes in total (which he identifies as *n*). The example, however, is a singular choice, since produces the same results as the 'incorrect' SV procedure.

The analyses of simulated networks provided by Chen suggest that there could be other serious issues in his implementation of the SV procedure. This is particularly evident in the analyses conducted on 'perfectly segregated' food webs, given that $\overline{\mathrm{N}}$ should be equal to -1 for all of them, while Chen shows this metric varying from -1 to 0 in his analyses (the same applies to Chen's analysis of perfectly nested food webs).

Chen further claims (incorrectly) that his equation and that of SV give very different quantification of node overlap in nested networks. Again, his error is possibly due to the same implementation problems we highlighted above. To demonstrate the basic equivalency of Chen's equation 6 and the SV method, we have replicated the analysis on the same set of food webs used by SV (Cohen 2010), using both the SV method and Chen's method (Python code for both SV's and Chen's implementation of $\overline{\mathrm{N}}$ is provided as Supporting Information). The two approaches produce very similar results (Fig. S2A; R^2 = 0.97; intercept = 0.04; coefficient = 0.95). We also found that the (always small) discrepancies between the two methods tend to further decrease with increasing network size (both in terms of nodes and edges) (Fig. S3). Thus, if we remove from the analyses very simple and unrealistic networks (i.e., those having less than 30 edges), we find that the two methods produce virtually indistinguishable results (Fig. S2B; R^2 = 0.999; intercept = 0.02; coefficient = 0.97). We have also compared the two methods on 1000 random (Erdős–Rényi) networks, finding, again, no appreciable difference (R^2 = 0.99; intercept = -0.004; coefficient = 0.98). The lack of a difference occurs because Equation 6 of Chen is unnecessarily complex; it gives the same probability as the SV probability equation except when $d1$, $d2$, k, and n are all very small (e.g., < 10) and thus represent an unrealistically (or uninterestingly) simple network. We also point out that Chen's Equation 6 cannot be solved when $k = d1$ or $k = d2$ because this would involve negative numbers in some of the combinatorics terms.

Finally, Chen's analysis on the effect of generalist species on the discrepancy between SV method and Chen's method raises serious doubts. Letting alone the lack of ecological realism in generating food webs with 50 nodes (we think Chen refers to this measure, when referring to size) and a connectance of 'around' 0.9 (i.e. almost fully connected graphs), we wonder how it is possible to create networks with fixed connectance and size, while varying the number of generalists (which Chen defines as nodes connected to "all the remaining species/nodes" in the network).

The SV method, although providing a summary statistic for a given network, relies on and summarizes pairwise comparisons between species and/or sites, as in the case of the popular NODF nestedness metric (Almeida-Neto *et al.* 2008). This provides it much freedom to test complex null hypotheses by identifying permitted and forbidden species interaction links according to different ecological criteria and on a per-species basis. Besides technical issues such as those we highlighted here, the identification of a 'universal' rule controlling all pair comparisons (as the *n* adjustment proposed by Chen) goes against this flexibility, that is where the true potential of our method lies.

Supplementary References

Almeida-Neto, M., Guimaraes, P., Guimarães, P.R., Loyola, R.D. & Ulrich, W. (2008) A consistent metric for nestedness analysis in ecological systems: reconciling concept and measurement. *Oikos*, **117**, 1227-1239.

Chen, Y. (2016) Newly proposed node segregation index is not suitable for analyzing unimode food webs. *Ecological Complexity*, ***26***, 89-94.

Cohen, J.E. (2010) *Ecologists' Co-Operative Web Bank. Version 1.1. Machine-readable data base of food webs.* The Rockefeller University, New York. URL http://hdl.handle.net/10209/306 [accessed 10 December 2014]

Fox, L.R. (1975) Cannibalism in natural populations. *Annual review of ecology and systematics*, 87-106.

Gross, J. L. & Yellen, J. (2005). *Graph theory and its applications*. CRC press.

Newman, M.E. & Park, J. (2003) Why social networks are different from other types of networks. *Physical Review E*, **68**, 036122.

Strona, G. & Veech, J.A. (2015) A new measure of ecological network structure based on node overlap and segregation. *Methods in Ecology and Evolution*, **6**, 907-915.

Supplementary Figures

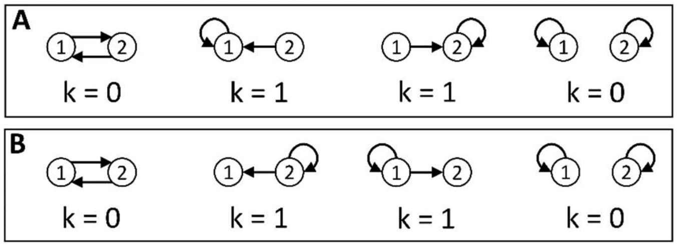

Fig. S1. Different configurations of the directed network in Chen's Fig. 2 not altering, respectively, out-degree (**A**) and in-degree (**B**). *k* indicates the number of shared nodes. Note that the average node overlap is equal to 0.5 in both cases.

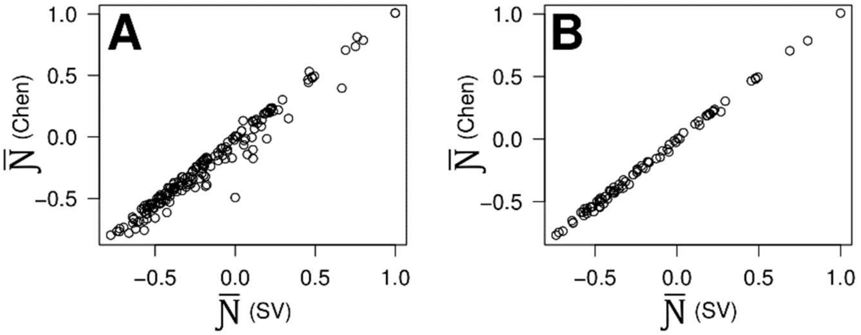

Fig. S2. Comparison between the original SV measure and Chen's modified formula on the set of food webs used as a benchmark in SV, available from the ECO-WeB data base (Cohen 2010). Panel **A** reports the results of all networks, while panel **B** reports the results for all networks having more than 30 edges.

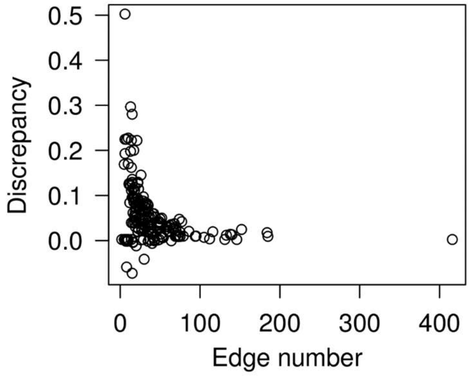

Fig. S3. The discrepancy (measured as difference) between SV's and Chen's implementations of $\overline{N}$ approaches rapidly 0 as network size increases.
